# Adrenal Adenoma Anarchy: A Case of an ACTH-Secreting Pheochromocytoma

**DOI:** 10.1155/2020/4869467

**Published:** 2020-02-26

**Authors:** Michelle N. Lee, WingYee Wan, Dianna C. Chormanski, Maria I. Kravchenko

**Affiliations:** ^1^Department of Medicine, Department of Endocrinology, San Antonio Military Medical Center, 3551 Roger Brooke Drive, JBSA Fort Sam Houston, San Antonio, TX 78234, USA; ^2^Department of Pathology, San Antonio Military Medical Center, 3551 Roger Brooke Drive, JBSA Fort Sam Houston, San Antonio, TX 78234, USA

## Abstract

*Introduction*. Pheochromocytomas are rare neuroendocrine tumors that arise from sympathetic adrenomedullary chromaffin tissue. Depending on the amount of catecholamines they secrete, they have variable presentations. There have been reported cases of adrenocorticotrophic (ACTH) secreting pheochromocytomas that present with severe Cushing syndrome. Here, we present a pheochromocytoma with adrenocorticotrophic hormone (ACTH) cosecretion, which due to its rarity and variable presentation, may be a diagnostic challenge. *Presentation*. A 64-year-old woman with history of colon cancer presented with new-onset diabetes, worsening hot flashes, and hypertension. On CT imaging she had an enlarging right adrenal nodule (1.7 cm) with 60 Hounsfield units of attenuation and no PET avidity. Biochemical evaluation showed elevated urinary and plasma metanephrines, elevated plasma cortisol levels despite dexamethasone suppression, elevated late-night salivary cortisol, and high-normal adrenocorticotrophic hormone. The patient underwent laproscopic right adrenalectomy, and pathology confirmed pheochromocytoma. Her lab abnormalities and symptoms of hot flashes and hypertension improved postoperatively. *Conclusion*. This case demonstrates an unusual ACTH-secreting pheochromocytoma with subtle presentation and highlights the importance of obtaining a complete biochemical evaluation of incidental adrenal adenomas.

## 1. Introduction

Pheochromocytomas are rare neuroendocrine tumors that arise from sympathetic adrenomedullary chromaffin tissue. Presentations are variable depending on the amount of catecholamine secretion. Classically, patients present with the nonspecific triad of palpitations, headaches, and/or sweating, although recent studies have found only 17% of patients diagnosed with pheochromocytoma to present classically [1]. Another study by Gruber et al. reported 61% of pheochromocytomas to be asymptomatic and diagnosed on incidental imaging. Due to its nonspecific presentation, a high level of suspicion is needed for the diagnosis of a pheochromocytoma and rare ACTH-producing pheochromocytomas may be a diagnostic challenge. Patients can present with severe Cushing Syndrome (CS), resistant hypertension, diabetes mellitus, and hypokalemia. Due to the high prevalence of obesity, metabolic syndrome, diabetes mellitus, and hypertension, and the potentially subtle physical findings associated with cortisol excess, the diagnosis of CS alone can be challenging. Here, we report how an ACTH-secreting pheochromocytoma, being a rarity, can cause a delay in diagnosis and treatment.

## 2. Case Presentation

**Figure 1:**
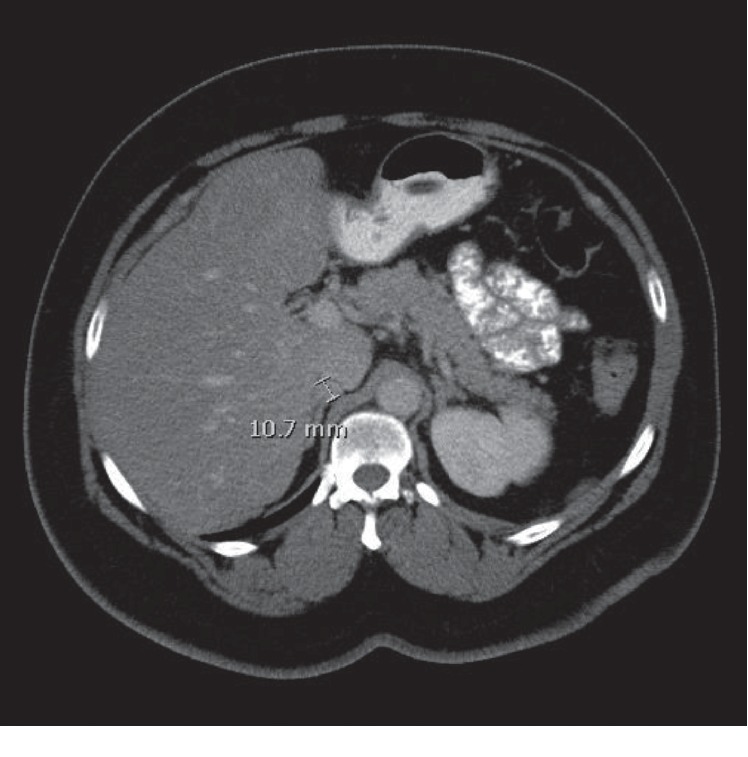
Initial CT imaging of incidental right adrenal nodule measuring 1.1 cm.

**Figure 2:**
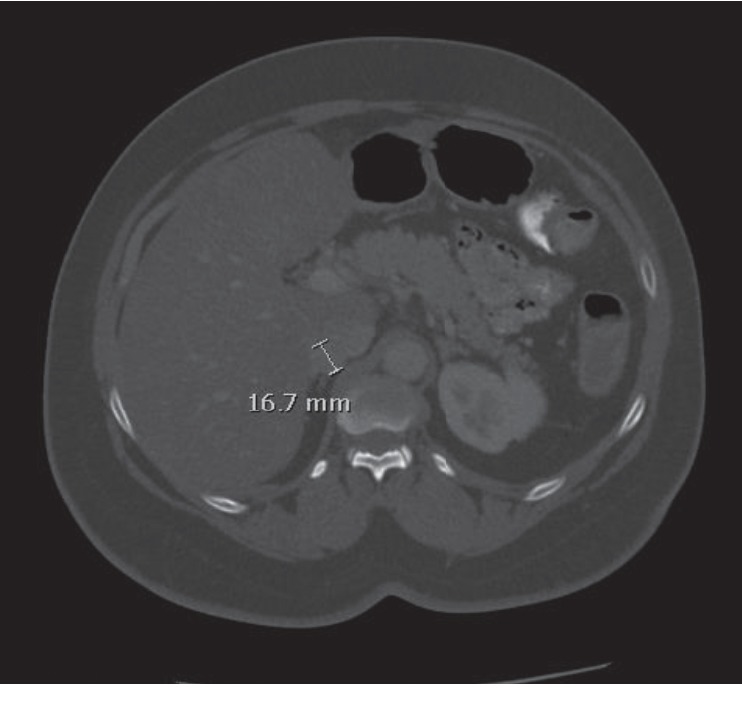
Follow-up CT noting an increase in size of the right adrenal nodule (1.7 cm) with 60 Hounsfield units of attenuation (right).

A 64-year-old woman with a history of hypertension, obesity, diabetes mellitus, and ileocecal carcinoma status post right hemicolectomy with ileal resection was evaluated in 2018 for hot flashes, worsening hypertension, and new-onset diabetes in association with an enlarging adrenal nodule. Physical exam was notable for an elevated blood pressure to 165/93 but did not reveal abdominal striae, central obesity, or hirsutism. She had a history of a 1.3 cm right adrenal nodule on CT abdomen/pelvis as early as 2012, found incidentally on evaluation for her colorectal cancer. It was not further evaluated until 2013, at which time it was stable in size (Figure 1). Her hormonal workup in 2013 was remarkable for elevated urine metanephrines to less than twice the upper limit of normal, an appropriately suppressed cortisol level on her overnight dexamethasone suppression test (DST), and normal renin and aldosterone levels (Table 1). The patient was again lost to follow-up for several years and continued to have poorly controlled hypertension and worsening diabetes mellitus. On surveillance CT in October 2018, the adrenal nodule was noted to have grown to 1.7 cm in size with 60 Hounsfield units of attenuation and no PET avidity (Figure 2). Repeat laboratory evaluation revealed elevated urinary metanephrines and elevated serum metanephrines, now three to four times the upper limit of normal (free metanephrines 403 pg/mL (0–62), free normetanephrine 482 mcg/mL (0–145)). She now had a nonsuppressed cortisol on DST (cortisol 11.4mcg/dL (0–3), dexamethasone 226 ng/dL), two elevated late night salivary cortisol readings (0.173 mcg/dL, 0.097 mcg/dL (0–0.090)), and a high-normal ACTH level of 45.62 pg/mL (6–50) (Table 1). The patient's serum potassium was consistently normal.

**Figure 3:**
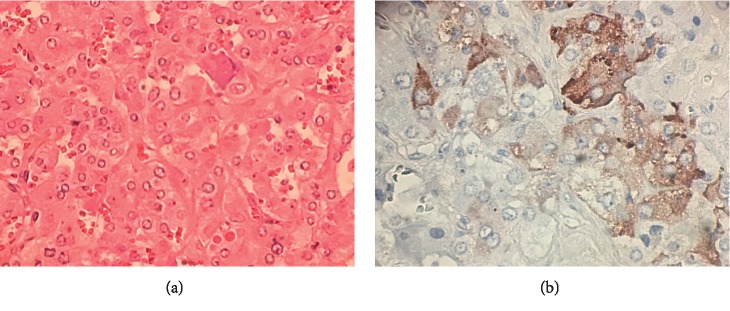
(a) Hematoxylin and eosin stain demonstrating polygonal tumor cells with vesicular nuclei and some prominent nucleoli growing in a nested pattern. (b) Immunohistochemical stain for adrenocorticotrophic hormone demonstrating tumor cells with patchy positive staining (brown).

The patient was diagnosed with an ACTH-producing pheochromocytoma, and due to the lack of PET avidity, there was less concern for colon cancer metastasis. Doxazosin and a high salt diet were initiated prior to undergoing an uncomplicated laparoscopic right adrenalectomy in May 2019. Pathology confirmed pheochromocytoma with low mitotic index and positive for synaptophysin and chromogranin. Immunohistochemical staining confirmed ectopic hypersecretion of ACTH (Figures 3(a) and 3(b)).

Her hot flashes, hypertension, and diabetes significantly improved following surgery, and she no longer required doxazosin or insulin. Repeat laboratory testing showed normalization of her cortisol on DST (cortisol 0.4 mcg/dL, dexamethasone 240 ng/dL), normal ACTH (42 pg/mL), normal metanephrine levels (35 pg/mL), and only slightly elevated normetanephrine levels (189 pg/mL), deemed normal for her age and history of essential hypertension.

## 3. Discussion

Pheochromocytomas, although rare, are becoming more common due to incidental detection on CT. Gruber et al. identified that incidental pheochromocytomas were typically found in older patients, with fewer symptoms and with a smaller degree in elevation of urine and serum metanephrines compared to those diagnosed based on symptoms. Incidentally discovered pheochromocytomas were also found to require less cumulative phenoxybenzamine compared to those with symptoms [2].

This patient's pheochromocytoma was initially identified as an incidentaloma on multiple CT scans for surveillance of her colon cancer. Her symptoms of worsening hypertension and night sweats were not the classic triad presentation for a pheochromocytoma, which may be due to having only moderate catecholamine secretion. There was also a concern for subclinical Cushing's syndrome based on her nonsuppressed cortisol on DST and elevated late night salivary cortisol levels.

ACTH-producing pheochromocytomas are rarely diagnosed, comprising approximately 5% of cases of ectopic ACTH syndrome, with less than 100 reported cases [3]. It more commonly presents in women and unilaterally. Patients will typically present with severe CS with diabetes mellitus and significant hypokalemia and will less likely present with catecholamine excess [4]. The diagnosis can sometimes be challenging as symptoms are nonspecific, although they typically present less insidiously than classic Cushing's syndrome. Our patient's presentation was more insidious, without clinical symptoms or signs of Cushing's syndrome or hypokalemia that would suggest a secondary cause of her hypertension and diabetes mellitus. Hypertension and intermittent sweating or hot flashes were her only symptoms of catecholamine excess.

Patients with ectopic ACTH syndrome can present with variable ACTH as well, ranging from normal levels, as seen in our patient, to over 200 pg/ml [3, 5]. Although her preoperative ACTH was within normal limits, this was inappropriate in the setting of her hypercortisolism. Repeat ACTH postoperatively was slightly improved and appropriate for a preserved hypothalamus-pituitary-adrenal axis. Due to already having a diagnosis of a right pheochromocytoma, the decision to undergo an adrenalectomy was made prior to any further workup for hypercortisolism. The normal postoperative suppression of cortisol and positive ACTH staining of the tumor confirmed that her pheochromocytoma was the ectopic source. If further workup was necessary, for example, if ACTH staining was negative, the tumor could have been stained for CRH to assess for ectopic secretion. Additionally, a high dose DST or CRH stimulation test could assist in differentiating a pituitary from an ectopic source. High-dose DST is noted to have only 76.5% accuracy, as 20–30% of ectopic ACTH-producing tumors have functioning glucocorticoid receptors that can still suppress ACTH production [4].

It is important to treat the excess catecholamines and cortisol prior to surgery in ACTH-producing pheochromocytomas. Patients are typically treated with an alpha blocker in addition to other antihypertensives to control their hypertension. In the case of the hypercortisolism, treatment is dependent on the severity and may include metyrapone and/or ketoconazole to directly prevent further production of cortisol, potassium supplementation to correct hypokalemia, and insulin to control hyperglycemia. Medical therapies are usually tapered and discontinued following adrenalectomy. Patients with a unilateral adrenalectomy will typically still require a short course of steroids [3]. As our patient did not present with overt Cushing's syndrome, preoperative ketoconazole was not deemed necessary.

Unlike other ectopic ACTH-producing tumors, pheochromocytomas are usually benign and cured following unilateral adrenalectomy. Many other tumors that have been associated with ectopic ACTH production are usually metastatic at the time of diagnosis and may require palliative bilateral adrenalectomy [5, 6].

## Figures and Tables

**Table 1:** Hormonal evaluation.

	Initial screening Jul 2013	Repeat screening Nov 2018	Jan 2019	Jun 2019 (1 month postoperative)
AM cortisol with 1 mg overnight DST	1.2 mcg/dL	7.3 mcg/dL	11.4 mcg/dL	0.4 mcg/dL

Late-night salivary cortisol at 0300 (0–0.090 mcg/dL)			0.175 mcg/dL, 0.173 mcg/dL, 0.097 mcg/dL	

24 hr urinary cortisol (36–137 mcg/24 hr)			89.0 mcg/24 hr	

Adrenocorticotrophic hormone (ACTH) (6.00–50 pg/mL)			45.62 pg/mL	

24 hr urinary metanephrines (74–297 mcg/24 hr)	505 mcg/24 hr	864 mcg/24 hr		

24 hr urinary normetanephrine (105–354 mcg/24 hr)	434 mcg/24 hr	690 mcg/24 hr		

24 hr urinary metanephrines, total (179–651 mcg/24 hr)	939 mcg/24 hr	1554 mcg/24 hr		

Plasma-free metanephrine (0–62 pg/mL)	—	403 pg/mL	199 pg/mL	35 pg/mL

Plasma-free normetanephrine (0–145 pg/mL)	—	482 pg/mL	176 pg/mL	189 pg/mL

Plasma renin (0.25–5.82 ng/mL/h)	1.77 ng/mL/h	1.121 ng/mL/h	—	—

Plasma aldosterone (0–30 ng/dL)	13 ng/dL	10.6 ng/dL	—	—

## Abbreviations

ACTH: Adrenocorticotrophic hormone
CS: Cushing syndrome
DST: Dexamethasone suppression test
CRH: Corticotropin-releasing hormone.
